# A taxing problem: The impacts of research payment practices on participants and inclusive research

**DOI:** 10.1371/journal.pone.0303112

**Published:** 2024-06-06

**Authors:** Leslie E. Wolf, Samantha Kench, Christy J. W. Ledford

**Affiliations:** 1 Georgia State University College of Law, Atlanta, Georgia, United States of America; 2 Department of Family and Community Medicine, Medical College of Georgia at Augusta University, Augusta, Georgia, United States of America; Tehran University of Medical Sciences, ISLAMIC REPUBLIC OF IRAN

## Abstract

Empirical data regarding payments to participants in research is limited. This lack of information constrains our understanding of the effectiveness of payments to achieve scientific goals with respect to recruitment, retention, and inclusion. We conducted a content analysis of consent forms and protocols available on clinicaltrials.gov to determine what information researchers provide regarding payment. We extracted data from HIV (n = 101) and NIMH-funded studies (n = 65) listed on clinicaltrials.gov that had publicly posted a consent form. Using a manifest content analysis approach, we then coded the language regarding payment from the consent document and, where available, protocol for purpose and method of the payment. Although not part of our original planned analysis, the tax-related information that emerged from our content analysis of the consent form language provided additional insights into researcher payment practices. Accordingly, we also recorded whether the payment section mentioned social security numbers (or other tax identification number) in connection with payments and whether it made any statements regarding the Internal Revenue Service or the tax status of payments. We found studies commonly offered payment, but did not distinguish between the purposes for which payment may be offered (i.e., compensation, reimbursement, incentive, or appreciation). We also found studies that excluded some participants from receiving payment or treated them differently from other participants in the study. Differential treatment was typically linked to US tax laws and other legal requirements. A number of US studies also discussed the need to collect Social Security numbers and income reporting based on US tax laws. Collectively, these practices disadvantage some participants and may interfere with efforts to conduct more inclusive research.

## Introduction

Payments to research participants are common for both pragmatic and ethical reasons [1–4]. Payments facilitate recruitment and retention [4,5] and, thus, ability to answer scientific questions that justify exposing participants to research risks [6]. They demonstrate respect for the participants who contribute their time and energy to research efforts, generally to benefit others [4,7]. Such payments also minimize financial risks to participants by defraying the costs of participation when reimbursing out-of-pocket costs, such as travel, and providing compensation for the time required by the research [4,7,8]. In this way, payments can be seen as advancing core research ethics principles of respect for persons, beneficence, and justice [9].

Ethical concerns have long been raised that payments to research participants may compromise consent by exerting undue influence, particularly when participants are considered “vulnerable,” including “economically or educationally disadvantaged persons” [10]. IRBs also express concerns that payments may increase harms to participants, if, for example, they are used to purchase drugs or alcohol [11,12]. More recently, commentators and the Secretary’s Advisory Committee on Human Research Protections (SACRHRP) have opined that most types of research payments do not raise undue influence concerns [13,14]. They argue that payment not only is ethically permissible but may be ethically required [4,13,15]. Payments that offer reimbursement and fair compensation are not only respectful, but can ameliorate the differential burdens on research participants of different backgrounds, minimize financial risks, and produce more inclusive, just research. But, as commentators have recently pointed out, the fairness of research payments are affected by tax policies [8,16]. For example, because tax rates differ by state, participants in the same trial could effectively receive different payments, violating conceptions of justice, and, because the US Internal Revenue Service (IRS) treats research payments as income, such payments could jeopardize participants’ access to other benefits and, thus, health and financial well-being [8].

Despite the perceived importance of participant payment to successful completion of research and the ethical debate about payments, empirical data regarding research participant payments is limited [1,2,7,17–19]. We previously conducted a content analysis of published articles in four journals to assess how often and how researchers disclose payment information when reporting research results [20]. We found that few (<6%) of the articles included information about payment types or structure. This lack of information limits our understanding of the effectiveness of payments in terms of achieving scientific goals with respect to recruitment, retention, and inclusion [20]. Given payment information must be included in consent forms and likely is included in research protocols, to fill in this gap, we conducted a content analysis of consent forms and protocols available on clinicaltrials.gov.

## Methods

Given our interest in vulnerable populations and the expressed concerns about research payments to educationally and economically disadvantaged individuals being potentially coercive [2,13,20] and payments being used to purchase drugs or alcohol [11,12], we sampled studies on HIV/AIDs and studies funded by the National Institute of Mental Health (NIMH) [21,22]. We chose to sample HIV/AIDS studies because, in the United States, people at risk for or living with HIV/AIDS are disproportionately members of historically marginalized groups and economically disadvantaged [23,24]. Substance use is an important risk factor HIV/AIDS [21,22]. We chose to sample NIMH funded studies because mental health remains highly stigmatized [25,26] and is similarly associated with economic disadvantage and historical disadvantage [27,28]. In addition, NIMH’s research portfolio includes HIV-related research and research on mental health disparities [29].

This study was conducted, in part, as a pilot to understand the information available on clinicaltrials.gov. Accordingly, our inclusion criteria were broad. Studies were eligible for inclusion if they 1) were an HIV-related clinical research study (including social/behavioral research) and NIH funded OR funded by NIMH, 2) had completed enrollment, and 3) had a consent document available on the clinicaltrials.gov website. A research team member (SK) conducted searches using the advance search function on clinicaltrials.gov (now classic.clinicaltrials.gov) to identify eligible studies. Specifically, for HIV-related clinical research studies, SK input “HIV” into the text box labeled “Condition or disease”, selected “completed” under “Status: Recruitment,” and selected “Informed Consent Forms (ICFs)” under “Study documents”. The searches for NIMH-funded research studies was conducted similarly, although “National Institute of Mental Health” was entered into under Targeted Search: Sponsor/Collaborator, instead of specifying “HIV” for “Condition or disease”. SK’s available effort required that the searches be conducted sequentially.

Searches for HIV clinical research studies were conducted between August 2022 through October 2022 and for NIMH-funded clinical research studies between January 2023 through February 2023. Because NIMH funds HIV-related research, the HIV clinical research studies and the NIMH-funded clinical research studies results had some overlap. We cross-checked the lists and removed 21 duplicate studies from the list of NIMH-funded studies. We included both domestic and international studies. (See S1 Dataset)

For each eligible study, the following data were extracted and recorded in an Excel file: study name, study completion date, available documents (consent form, study protocol, or both), whether participants were offered payment, the amount of any payment, the payment method, and the clinicaltrials.gov identifier.

The information on payment amount and method was copied verbatim from the study consent document and, where available, protocol. A study team member (SK) read the full documents to identify this information. To ensure payment information was captured completely, SK also conducted searches within the documents for keywords (“payment”, “renumeration”, “compensation”, “consideration”, “reimburse”), although some document formats did not permit searching (PDFs contained pictures of documents instead of text).

Using a manifest content analysis approach [30], we then coded the language regarding payment from the consent document and, where available, protocol for purpose and method of the payment. Regardless of the language the researchers used, consistent with the SACHRP approach [13], we classified the payments to participants as follows. Payments for out of pocket costs, such as parking and other transportation costs, were classified as *reimbursements*. Payments for participants’ time, whether explicitly by the expected time to complete a study task or by visit, were classified as *compensation*. Payments designed to encourage behavior to advance research goals, such as completion of tasks within a certain time period or referral of eligible participants, and provide a net benefit to participants (i.e. beyond reimbursement or compensation) were classified as *incentives*. Non-monetary items were classified as *appreciation*. To provide a complete picture of payments and other items participants may receive, we also noted payments or other items (e.g., food) that did not fall within these categories. See Table 1 for illustrative examples of each payment type. For method of payment, when specified, we captured whether payment was made in cash, by debit or gift card, by Clincard (a specific type of debit card), check, voucher, or other. Although not part of our original planned analysis, the tax-related information that emerged from our content analysis of the consent form language provided additional insights into researcher payment practices. Accordingly, we also recorded whether the payment section mentioned social security numbers (or other tax identification number) in connection with payments and whether it made any statements regarding the Internal Revenue Service or the tax status of payments.

**Table 1:** Payment types.

Type of payment	Operational Definition	Sample language
Reimbursement	Payments for out of pocket costs (e.g., parking, transportation)	“You will be reimbursed $25 to $100 for parking and travel-related expenses for each scheduled study visit depending on the distance traveled from your residence to the clinic.” (NCT02121756)
Compensation	Payments for participants’ time (i.e. task or visit)	“You will be compensated $40 for your time and effort for each of the 11 scheduled study visits that you complete.” (NCT04302896)
Incentives	Payments to encourage behavior to advance research goals (e.g., task completion within time period, referral of participants)	“Because it is very important for the research to get the surveys completed on time, we will provide you 2 bonus.. the 1^st^ when you complete 3 or the 4 surveys and the 2^nd^ bonus when all 4 are completed.” (NCT03065517)
Appreciation	Non-monetary items	“As a token of appreciation, you will receive study related promotional items as you complete visits.” (NCT02711878)

## Results

### Mentions payment in consent form or protocol

#### HIV clinical research studies

We identified 101 HIV clinical research studies with completion dates ranging from 2008–2022, although 95% of the studies completed enrollment after 2016. The majority of these clinical research studies (92/101) had language regarding payment to participants, although 20 of these did so only to instruct individual sites to determine whether to offer payment and how much. Nine studies did not pay participants (5 explicitly state this, whereas 4 are silent on compensation).

#### NIMH-funded clinical research studies

We identified 65 NIMH-funded clinical research studies with completion dates ranging from 2011–2022; 99% of the studies completed enrollment after 2016. These studies included research among healthy volunteers, as well as a number of mental health conditions, including attention deficit hyperactivity disorder, bipolar disorder, depression, schizo-affective disorder, anxiety disorders, autism spectrum disorders, and eating disorders. All of these clinical research studies had some language regarding payment to participants, although 2 explicitly stated that participants would not be paid.

### Types of payment

*Compensation* (payments for study-related tasks). We found that studies commonly made compensation payments to participants (63/101 HIV studies; 58/65 NIMH-funded studies; 72% of all studies). These payments often were described as per visit or for completion of specific study procedures (e.g., screening, particular test, survey, etc.) (36/63 HIV studies; 37/58 NIMH-funded studies). Some studies described these payments as for participants’ “time and effort” (7/63 HIV studies; 9/58 NIMH-funded studies) or simply “time” (9/63 HIV studies; 6/58 NIMH-funded studies). Others described them as being for participants’ “time and inconvenience” (9/63 HIV studies; 1/58 NIMH-funded studies), whereas one HIV trial referred simply to “inconvenience” and one NIMH-funded trial described the payments as for “discomfort and inconvenience.” U.S. based studies (100/112, 89%) provided compensation more often than international studies (20/41, 49%).

*Reimbursement* (payments for out-of pocket costs). Studies also commonly reimbursed participants for their out-of-pocket costs (52/101 HIV studies; 19/65 NIMH-funded studies; 42% of all studies). More international studies (22/41, 54%) and studies including both international and U.S.-based sites (8/14, 62%) offered reimbursement than U.S.-based studies (41/112, 37%). Six of the HIV studies and two of the NIMH-funded studies (5% of all studies) limited payments to reimbursement (i.e., for travel expenses or other out of pocket costs). Costs of traveling to the research site, including parking, mileage, public transportation, and rideshare costs, were among the costs listed as being reimbursed by the studies. Lodging was also sometimes offered. Only two studies (1 HIV and 1 NIMH-funded) mentioned childcare as one of the costs for which they sought to reimburse participants. Studies typically provided a fixed amount in reimbursement, which may be bundled within the overall payment. For example, one study indicated, payment was “for their time, effort, and travel to and from the clinic.” (NCT02929131, HIV)

A few studies separated out the reimbursement payment from other payments, and a few reimburse different amounts for travel depending on distance. For example, one study indicated “To help with the cost of gas, for participants traveling > 20–40 miles one way for their appointments, they will also be given a $15.00 gas card to cover the cost of the transportation. Participants traveling > 40 miles one way for their appointments will also be given a $30.00 gas card to cover the cost of transportation.” (NCT02856269, HIV). Two (one HIV and one NIMH-funded trial) required receipts for reimbursing travel costs. For example, one study explained “We will also pay you back for travel costs of up to $50 per visit once your give us receipts and/or, if you use your own car, a record of the number of miles your drove. For... visits in…, we can arrange for a car service to transport you (instead of paying you back).” (NCT04366258, NIMH) Another explained “We will ask you to save your receipts for travel expenses and give the receipts to us so we can pay you.” (NCT03934541, HIV)

*Incentives* (payments that advance study goals (e.g., task completion within time period, referral of participants) and provide a net benefit to participants (i.e., beyond reimbursement or compensation). Incentive payments were considerably less common (11/101 HIV studies and 5/65 NIMH-funded studies). U.S. based studies (14/112, 13%) offered incentives more frequently than international studies (2/41, 5%). For the NIMH-funded studies, incentives were offered to encourage completion either of all the study procedures or certain procedures within a certain time period. For the HIV studies, incentives were offered for completing study procedures (11/101), the goals were more varied than the NIMH-funded studies (e.g., one for attending the first visit, another to non-responders), and three incentives were offered as a lottery, rather than a guaranteed payment. Four studies also provided incentives for referring participants who proved eligible for and enrolled in the study, and one study offered an incentive for returning a device used during the study.

*Appreciation* (non-monetary items). Only one HIV trial offered study-related promotional items, in addition to reimbursement and compensation payments. Three HIV studies mentioned refreshments being provided during study procedures, and two offered “food staples” that participants could take home with them. One HIV trial and four NIMH-funded studies offered other goods to participants (data, promotional items, HIV test, and toy).

There was no study that offered only appreciation payments.

*Other*. Three HIV studies and three NIMH-funded studies offered lotteries that created opportunities for additional money or items.

See Table 2 for a summary of the characterisics of included studies.

**Table 2:** Characteristics of included studies*.

	HIV studies (n = 101, 61%)	NIMH-Funded Studies (n = 65, 39%)	Total (n = 166)
**Payment Types**			
Compensation	63 (62%)	58 (89%)	121 (73%)
Reimbursement	52 (51%)	19 (29%)	71 (43%)
Incentives	11 (11%)	5 (8%)	16 (10%)
Appreciation	1 (1%)	0 (0%)	1 (1%)
Other	8 (8%)	7 (11%)	15 (9%)
**Payment Methods**			
Did Not Specify	49 (49%)	29 (45%)	78 (47%)
Debit Card/Gift Card or Certificate/Clincard	21 (21%)	28 (43%)	49 (30%)
Cash	12 (12%)	3 (5%)	15 (9%)
Check	3 (3%)	5 (8%)	8 (5%)
Voucher	6 (6%)	5 (8%)	11 (7%)
Other^+^	6 (6%)	5 (8%)	11 (7%)
**Geographic Location**			
US-Based	55 (55%)	57 (88%)	112 (68%)
International	33 (33%)	7 (11%)	40 (24%)
Both US-Based and International	13 (13%)	0 (0%)	13 (8%)
**Taxation Information**			
Mentioned SSN	18 (18%)	11 (17%)	29 (17%)
Mentioned IRS/Taxation	21 (21%)	9 (14%)	30 (18%)

*Percentages may not total to 100% because of rounding and studies may use multiple payment types and methods.

^+^ “Other” payments included promotional items, other tangible goods (including food and other household staples), points available for use at the institution as an alternative, transportation card, direct deposit (for employees).

### Explanation of payment

Some statements about the purpose of the payments were explicit. For example, multiple studies connected the payment to the participants’ time: “We will give you $15 to thank you for your time” in the consent form. (NCT0169789, NIMH); “The gift certificates are to pay for time spent completing the evaluations.” (NCT01201382, NIMH); “This is to compensate you for your time.” (NCT02494817, HIV) Similarly, some studies were explicit that they were offering an incentive: “In order for this project to have scientific value, we need to know whether our interventions were helpful. Therefore, we will make every effort to stay in touch, and we will compensate your child an additional $5 gift card if you or your child lets us know about any changes in your/their contact information.” (NCT02332239, NIMH); “Because it is very important for the research to get the surveys completed on time, we will be able to provide 2 bonuses.... You will get the 1^st^ bonus when you complete 3 out of the 4 surveys and the 2^nd^ bonus when all 4 surveys are completed.” (NCT03065517 & NCT03321617, NIMH) Finally, the sole appreciation payment is explicit as to purpose: “As a token of appreciation, you will receive study related promotional items as you complete visits.” (NCT02711878, HIV).

This explicit description also applied to multiple purposes. For example, one study explained in the protocol that “Monetary reimbursement for each interview and focus group meeting will help compensate subjects for their time and effort expended... and help defray any needed transportation or childcare expenses.” (NCT01890226, NIMH) Another indicated participants would be paid “for their time, effort, and travel to and from the clinic.” (NCT02939131, HIV) While these examples did not distinguish how much was received for what purpose, some studies did make these distinctions. For example, one study explained, “You will be provided with a voucher for [institution] parking at each visit. You will also be given a $10 stipend at each visit to cover time and other related expenses of participation.” (NCT 03888391, NIMH).

Some descriptions also specifically explained why researchers had chosen to limit payments to reimbursement: “We are aware of the ethical considerations in providing participant compensation to people who may be living in poverty because providing case payments can unintentionally coerce participation in research. Thus, we restrict compensation to cover the basic costs of participation by providing a travel reimbursement at each study visit.” (NCT04068688, NIMH (international)).

Yet, the payment descriptions in both the consent forms and protocols reflected confusion regarding what terms to use for payments and were, at times, contradictory. For example, one study told participants that they would receive *compensation* in a specific dollar amount when they were reimbursing for costs: “this *compensation* includes the parking fees that you might need to use while visiting us at [institution].” (NCT03033784, NIMH, emphasis added) Another described all participants receiving “a gift card as an *incentive*” for completing study tasks (NCT03478501, NIMH, emphasis added) Another explained their payment decision in the protocol as follows: “in order to make participation worth the subjects’ time, they will be *reimbursed* [amount].. . We have found this amount provides a good *incentive* to encourage ongoing participation.” (NCT04010461, NIMH, emphasis added) Another study suggested the following language: “We will give you [Site: insert *compensation*] for each study visit you complete. This amount is to cover the *costs* of [Site: insert text].” (NCT02404311, HIV, emphasis added (international)) Another explained “Compensation is provided to help cover travel expenses, as well as child care and time lost from gainful employment.” (NCT02588586, HIV, emphasis added) Yet precision was found, as one study notes, “Participants will not be *compensated*, however, they will be *reimbursed* for travel expenses up to $10 per visit.” (NCT01973283, NIMH) Another study broke down payments, explaining: “Research subjects will be offered $25 per hour up to a maximum of $150 for their participation in this study... You will be *reimbursed* [emphasis added] for gas at the rate of 55.5 cents per mile. Meals will be *reimbursed* [schedule][also provision for rideshare reimbursement and hotel].” (NCT03958903, NIMH, emphasis added).

This confusion regarding language sometimes resulted in contradictory statements. For example, one study informed participants, “You will receive *no money* for your or your baby’s participation in the study, but you *will be given money* for transportation costs to and from the clinic and to compensate you for your time." (NCT01229761, HIV, emphasis added (international)) Another study explained, “We will reimburse you the costs of public transport to the clinic on your appointment days.. There are no plans to give you money for taking part in this study.” (NCT00383669, HIV (international)) Another example included, “You will not be offered payment for being in this study. However, you will be reimbursed [amount] for your time and transport costs.” (NCT03054051, HIV (international)).

### Method of payment

Almost half of the studies did not specify in what form payments would be made (49/101 HIV studies; 29/65 NIMH-funded studies; 47% of studies offering payment). Of those that did, the most common option was to use a debit or gift card (21 HIV studies and 28 NIMH-funded studies (we included three NIMH-funded studies that specified a “gift certificate” rather than a gift card) or 30% of studies offering payment). Some studies specified the type of gift card; these included Amazon (3 HIV studies; 6 NIMH-funded studies), Visa (2 HIV studies; 2 NIMH-funded studies), and Target (1 HIV trial; 1 NIMH- funded trial). One HIV trial specified a phone card and another a gas card, without specifying a brand. In addition, 4 HIV studies and 2 NIMH-funded studies specified that they would use ClinCard, a special type of debit card. As one study explained, “The [Clincard] card scheme is run by Greenphire, an independent company specializing in payments for research studies and clinical studies.” Twelve HIV studies and 6 NIMH-funded studies (11% of studies offering payment) specified that they would provide participants with cash. U.S. based studies (41/112, 37%) offered payment by card more frequently than international studies (5/41, 13%). International studies (7/41, 18%) offered cash more frequently than U.S. based studies (11/112, 10%). As one such study notes, “You will be paid cash immediately after you complete the interview.” (NCT02815579, HIV (international)) Three HIV studies and 5 NIMH-funded studies paid participants with a check (including eChecks). Vouchers were offered by 6 HIV studies (for parking (3), meal (1), HIV test (1), and unspecified (2)) and 5 NIMH-funded studies (all for parking). Two HIV studies offered direct deposit, and two offered the option of goods instead of cash. One NIMH-funded study offered points as an alternative to cash for use on campus.

Risks of using a bankcard were identified by a few studies. For example, one study that offers a bankcard as one option indicated that “If you receive payment using a bankcard, the bank will have access to identifiable information. The bank will not have access to any medical information.” (NCT01948167 & NCT03928028 & NCT04045132, NIMH) These risks were expanded on in the following lengthy explanation from another study:

“You will receive compensation through a reloadable debit card, which will be provided to you. This “reloadable” card is NOT anonymous. For these cards, we would register you with the vendor of the debit cards, Bank of America (BOA) by providing your name, address, and phone number. You would receive one card, which would be “reloaded” after each completed study visit. This card can be used as a credit card OR as an ATM card. You would be able withdraw money from a bank either through a bank teller or an ATM machine. Please note that banks may charge a fee for using their ATM machine. If you lose the card, you can contact BOA and have the card replaced. Please read the information sheet that comes with the card for more information. This amount is to cover the costs of time and travel.” (NCT03928821, HIV)

See Table 3 for a summary of payment types and methods of payment.

**Table 3:** Payment types and methods between international and US-based studies*.

		International (n = 41, 25%)	US-Based (n = 112, 69%)	US and International (n = 13, 8%)	Total (n = 166)
	**Payment Types**				
Compensation		20 (49%)	100 (89%)	1 (8%)	121 (73%)
Reimbursement		22 (54%)	41 (37%)	8 (62%)	71 (43%)
Incentives		2 (5%)	14 (13%)	0 (0%)	16 (10%)
Appreciation		0 (0%)	1 (1%)	0 (0%)	1 (1%)
Other		6 (15%)	9 (8%)	0 (0%)	15 (9%)
	**Payment Methods**				
Did Not Specify		19 (48%)	51 (46%)	8 (62%)	78 (47%)
Debit Card/Gift Card or Certificate/Clincard		5 (13%)	41 (37%)	0 (0%)	46 (28%)
Cash		7 (18%)	11 (10%)	0 (0%)	18 (11%)
Check		0 (0%)	8 (7%)	0 (0%)	8 (5%)
Voucher		3 (8%)	7 (6%)	1 (8%)	11 (7%)
Other^+^		3 (8%)	8 (7%)	0 (0%)	11 (7%)

*Percentages may not total to 100% because of rounding and studies may use multiple payment types and methods.

^+^“Other” payments included promotional items, other tangible goods (including food and other household staples), points available for use at the institution as an alternative, transportation card, direct deposit (for employees).

### Identification/Taxation

Eighteen of the HIV studies and eleven of the NIMH-funded studies explicitly mentioned participants’ social security number in connection with payment, while twenty-one of the HIV studies and nine of the NIMH-funded studies explicitly mentioned that such payments may be taxable and/or they may be reportable to the U.S. Internal Revenue Service (IRS). We found these statements only among studies that were operating in the U.S. (whether exclusively or coupled with international sites), where the IRS has jurisdiction.

Various rationales for collecting social security numbers were offered. For example, one trial explained the request “is a necessary step to track that cards are given only to study participants. It will not be used for any other purpose without your permission.” (NCT02938598, NIMH) Another trial indicated that “In order to process your payment, we may need to collect your social security number. This information will be kept in a separate location from your research data and will be destroyed once your reimbursements have been processed.” (NCT04728815, NIMH) One trial explained, “Payments may only be made to U.S. citizens, legal resident aliens, and those who have a work eligible visa. You may need to provide your social security number to receive payment.” (NCT03958903, NIMH) More commonly, studies explained the requirement to report income to the IRS. For example, one explained that “Because you are being paid for your participation, [institution] is required by the Internal Revenue Service (IRS) to collect and use your social security number (SSN) or taxpayer identification number (TIN) to track the amount of money we pay.” (NCT04386096, NIMH) Several studies referred to the income threshold imposed by the IRS, although there was some variation here. One stated “Please note that [institution] is required to complete form 1099 for any participant payments over $475.00.” (NCT02264860, HIV) Another explained that “we are required to report payments more than $599.00 to the Internal Revenue Service.” (NCT04929262, NIMH) Although most put the burden of reporting on the institution, some put the burden on the participants. For example, one indicated, "If you receive more than $600 in a calendar year you may be required to report the compensation to the Internal Revenue Service (IRS)." (NCT01852942, HIV) Another stated, “Federal tax law requires you to report this payment as income to the Internal Revenue Service.” (NCT04929262, NIMH) Another explained that: “All compensation is taxable income to the participant regardless of the amount.” (NCT04672798, NIMH)

Some of these explained the consequences of failing to provide a social security number or other identifier. For example, one stated: “As is required by the laws that apply to [institution], in order for you to receive a payment (i.e., check), you need to give the study staff either your Social Security number or your Alien Registration number and will be asked to complete a IRS W9. If you do not have either of these numbers or are not willing to complete the IRS W9, you may be in the study but will not receive any payment.” (NCT04366258, NIMH) Similarly, one study explained, “Due to payment restrictions, only participants who are U.S. Residents or Citizens are eligible for the gift card lottery. If selected for the lottery, a participant can decline the gift card for any reasons, and will not be prompted to further report on their citizen status.” (NCT04108429, NIMH) Another indicated that “Individuals who do not provide a social security number may still participate in the research, but the IRS requires that 28% of the payment be sent by the institution to the IRS for ‘backup withholding’; thus you would only receive 72% of the expected payment.” (NCT04672798, NIMH)

Only one study described potential risks to participants from the payment process: “There is a potential loss of confidentiality due to interactions with the Office of the Treasurer and a delay of receipt of payment.” (NCT03928821, HIV).

One study conducted by the federal government contained the following warning: “If you have an unpaid debt to the federal government, please be aware that some or all of your compensation may be automatically reduced to repay that debt on your behalf.” (NCT02771249, HIV).

## Discussion

We found that payments for research participation most commonly involved compensation for time and effort, whether stated explicitly or offered for completion of study visits or tasks. Reimbursement for out-of-pocket costs, primarily transportation costs, also was frequently offered. Incentives and appreciation payments were less common. But researchers did not regularly explain in the consent form or the protocol the breakdown between these different types of payments nor did they use the language with precision. This is perhaps not surprising as, in common usage, these terms–“payment,” “compensation,” “reimbursement,” and “incentive” (among others)–are often used interchangeably. However, this lack of precision may make it difficult for IRBs to conduct their regulatory assessment that risks are minimized and reasonable in relation to anticipated benefits and that participants have been adequately informed [10,31]. It may also interfere with participants’ understanding and, thus, compromise the ability to provide informed consent [31]. The risk of participant confusion was illustrated most clearly in the statements that simultaneously say that participants will not receive any money for their participation but they will receive certain money. (“You will receive *no money* for your or your baby’s participation in the study, but you *will be given money* for transportation costs to and from the clinic and to compensate you for your time." (NCT01229761, HIV, emphasis added (international)) This lack of clarity also impedes our ability to conduct research to assess how payments are used in research. We saw one example that stated goals of defraying expenses and compensating participants for time and effort, yet offered an amount that could not achieve those goals in the location. Additional data could help assess the fairness of payments and lead to more inclusive practices [20]. The Secretary’s Advisory Committee on Human Research Protections (SACHRP) and others have set forth the different payment types we used here to guide our analysis [4,13,14]. Greater consistency and transparency regarding payments could be achieved if NIH, IRBs, and journals required researchers to explain their payment plans using the SACHRP framework in their protocols, consent forms, and papers reporting results [18]. Such information should also be required for clinical trial registration such as clinicaltrials.gov.

Failing to use this framework has practical implications for research participants as well. As we found, a significant minority of clinical studies discussed the tax implications of research payments. Indeed, several of these described payments as taxable income that must be reported–either by the institution or by the participant to the IRS. However, reimbursement payments are not *income* and, thus, are not taxable [32,33]. Lumping reimbursement payments with compensation and/or incentive payments can lead to participants paying excess tax. Thus, breaking down payments into their purposes not only can create greater clarity, but could lead to approaches that are fairer to participants and minimize financial risks.

The statements researchers included in their consent documents and/or protocols about IRS obligations suggest there is confusion–or different interpretations–among institutions about what is required. We found instances in which the income thresholds triggering institutional reporting of income to the IRS differed both from each other and the IRS requirements. For example, one listed an amount that is $125 less than the IRS threshold. Time does not explain this discrepancy, as the threshold has been at $600 for almost 70 years [34]. Another listed the threshold as above $599, which is inaccurate and may have tax implications for participants. We also found instances of statements declaring “all compensation is taxable income,” but, in addition to the concerns about lumping reimbursements and compensation together, some participants may not earn enough to be subject to tax [35]. Yet, such a definitive statement may prompt unnecessary time and energy expended trying to comply with the guidance provided by the researchers. These examples suggest that institutions require clarification of their obligations and the tax implications for research participants.

Yet, even if there were consistency across institutions regarding taxation, these statements about taxation raise concerns about their impact on informed consent. They may detract from the main purpose of the consent process to enable participants to make an informed decision about whether to participate in research by adding to the length and complexity of the consent process [36–39]. Our recommendations to break down payment purposes could exacerbate that process by requiring additional explanation. Moreover, previous research has suggested that some participants’ concerns about research participation are stoked by reference to government access to information [40]. Making researchers seemingly an agent of the government could exacerbate distrust and undermine inclusivity in research.

These concerns could be avoided, however, were any monies received for research participation excluded for income tax purposes [8]. In that case, a simple statement that “these payments are not taxable” would suffice. As Rand et al. explain, there are both precedents and ethical justifications to support a change in policy to exclude research participation payments from taxable income [8]. Making this policy change could also resolve another ethically troubling practice we discovered–reducing or refusing payment to participants who lack a social security number or who are unwilling to provide that information. Although such approaches may be consistent with tax law [41], asking already vulnerable individuals to participate without receiving the compensation that other participants receive takes advantage of that vulnerability. Excluding research payments from income would eliminate the need to request social security numbers and, thus, minimize confidentiality risks as required by the regulations [10]. Such exclusion would also avoid this inequitable treatment we identified while also removing a potential barrier to more inclusive participation. It would also remove other legal and economic risks [8,42].

Numerous studies have demonstrated the impact that payments for research participation can have in facilitating important research. However, there are still gaps in our knowledge about how and why payments are used. From our analysis, we have identified a few ways that we could fill those gaps and have suggested changes that could lead to greater equity in payment and more inclusive research. Our analysis has some limitations. We focused on HIV clinical research studies and those funded by the NIMH. Clinical research studies in other areas may have different payment practices. We also used a particular framework for classifying research payments. This framework has been criticized as conceptually confusing [15], and it may not be consistent with how researchers and participants think about such payments. This limitation may be exacerbated in international settings where different experiences and perceptions may influence payment practices and how payments are described or translated [43,44] and may explain the differences reflected in Table 3. Nevertheless, these data provide an important starting point for further research on research payment and ways to make such payments more equitable.

## Supporting information

S1 Dataset HIV-related and NIMH-funded studies. (XLSX)
